# Fracture resistance of endocrowns produced by 3D printing and CAD-CAM blocks: a comparative assessment

**DOI:** 10.1007/s00784-026-06780-4

**Published:** 2026-02-18

**Authors:** Kübra Nur Hekimoğlu, Salih Düzgün, Hüseyin Sinan Topçuoğlu

**Affiliations:** Department of Endodontics, Faculty of Dentistry, Erciyes University, 38039 Kayseri, Türkiye

**Keywords:** Endocrown, CAD-CAM, 3D printing, Fracture resistance

## Abstract

**Introduction:**

This study aimed to compare the fracture resistance of leucite reinforced glass ceramic (LRC), resin nanoceramic (RNC), lithium disilicate ceramic (LDS) blocks, and printable resin (PRM) used in the production of ferrulated and non-ferrulated endocrowns for mandibular molars.

**Methods:**

A total of 108 extracted mandibular molars with homogeneous dimensions and free of fractures, cracks, or caries were included. Root canal treatment was performed using the ProTaper Next rotary file system, and canals were obturated with gutta-percha and epoxy resin sealer by the lateral compaction technique. Specimens were divided into ferrule (F; 1-mm ferrule height), non-ferrule (NF), and control groups. Digital impressions were obtained with an intraoral scanner (Cerec Primescan), and restorations were designed in CAD software (inLab CAD SW 22.4). Based on restorative materials, groups were classified as follows: LRC, RNC, LDS, and PRM for both F and NF groups, in addition to a control group of intact teeth. Restorations were fabricated either by milling (LRC, RNC, LDS) or 3D printing (PRM), and surface conditioning was performed according to manufacturers’ instructions. All restorations were cemented using a universal adhesive system and dual-cure resin cement.Fracture resistance was tested in a universal testing machine (Instron, USA) at a crosshead speed of 1 mm/min until fracture occurred, and failure modes were classified as Type I (restoration fracture), Type II (restorable fracture involving tooth and restoration), or Type III (non-restorable fracture of tooth structure). Statistical analyses were performed using SPSS v23, with significance set at *P* < .05.

**Results:**

Fracture resistance values varied significantly among restorative materials (*P* < .05); intact teeth exhibited the highest resistance, while LRC restorations showed the lowest. PRM, RNC, and LDS restorations demonstrated significantly higher resistance than LRC, and all endocrown groups exceeded maximum masticatory forces reported in the literature. Comparisons between ferrule and non-ferrule groups fabricated from the same material revealed no significant differences (*P* > .05). Although failure type distribution was not significantly different among groups, LRC restorations were mostly associated with type I fractures, RNC and PRM exhibited predominantly restorable fractures, and LDS showed the highest incidence of catastrophic failures.

**Conclusion:**

The RNC, PRM, and LDS groups showed higher fracture resistance than LRC, and while LRC and RNC/PRM mainly resulted in restorable fractures, LDS was associated with more catastrophic failures.

**Clinical relevance:**

Endocrowns used Leucite Reinforced Ceramic showed the lowest fracture resistance. In contrast, the fracture resistance values of all materials used are greater than the physiological chewing force value. At the same time, the presence of a ferrule did not affect the fracture resistance values.

## Introduction

The restoration of endodontically treated teeth, which are affected by a higher risk of biomechanical failure, remains a challenge [1]. The conventional method involves using a crown supported by a post and core. However, this procedure may compromise the mechanical integrity of the tooth and increase the risk of root fracture [1]. With the improvements in adhesive dentistry, more conservative options have become popular, reducing the need for conventional post-and-cores in teeth [2]. Minimally invasive techniques are optimal treatment for endodontically treated teeth, aiming to preserve as much of the tooth structure as possible [3]. Endocrown is a restoration design based on the principle of minimally invasive dentistry for endodontically treated teeth [2, 4]. Endocrown restorations are monoblock restorations that achieve macromechanical retention from the pulp chamber and micromechanical retention through adhesive cementation [1, 5–7]. Endocrowns are particularly recommended for short, curved, calcified, or structurally damaged roots in molar teeth and in cases with significant coronal tissue loss or limited interocclusal space. Advances in CAD/CAM technology have led to the increased preference for endocrowns by enabling the rapid and accurate fabrication of precision restorations [8].

The introduction of new digital technologies related to 3D imaging, computer design, modeling, manufacturing, and material science has deeply influenced dentistry over the past few decades [9]. CAD-CAM systems consist of a scanner, software for processing scanned data, and a manufacturing unit that produces the restoration. This digital workflow enables clinicians to evaluate preparations and plan treatment by capturing both arches. It reduces chairside time and minimizes potential errors associated with conventional workflows, allowing same-day restoration delivery [10]. These advancements have resulted in significant time savings by enabling digital designs to be transferred directly to the production phase, which can be accomplished either through the milling of prefabricated blocks (subtractive method) or by using three-dimensional (3D) printers with additive manufacturing techniques [10]. In this way, the digital workflow streamlines clinical procedures while also improving the quality and speed of restorations. The subtractive system, as a well-established technology, offers the advantage of producing restorations from homogeneous materials that are not affected by environmental conditions [11]. Currently, many manufacturers favor the use of prefabricated blocks for fabrication processes utilizing subtractive manufacturing systems. While material waste generated during subtractive manufacturing cannot be reused, additive manufacturing minimizes material wastage.

In recent years, as the cost of 3D printing has decreased, the use of this technique in dentistry has become increasingly widespread [12]. Various additive manufacturing (AM) technologies are available today, including binder jetting, material extrusion, material spraying, vat polymerization, direct energy deposition, and powder bed fusion [11]. Among these, vat polymerization—comprising stereolithography (SLA) and digital light processing (DLP)—is the most widely used AM technology in dentistry. In SLA, an ultraviolet (UV) laser beam polymerizes and solidifies the photocurable resin layer by layer, whereas in DLP, a digital projector simultaneously cures each layer using projected light patterns. These technologies enable the production of highly accurate dental restorations with good surface quality and adaptation [13]. These advances in AM technologies have enabled the development of novel printable hybrid materials for definitive restorations.

Resin-based, printable hybrid materials (PRM) have been developed as an alternative to millable resin materials [9]. Ceramic-reinforced composites, such as resin nanoceramics, resin matrix glass ceramics, and printable resin materials, combine the mechanical and esthetic advantages of both ceramic and composite materials [9, 14]. Resin-based materials vary significantly in composition, including resin matrices, filler type, and ingredients [14]. There are studies comparing these resin materials, which can be used with different production methods [15–17].

There are many studies in the literature comparing the mechanical properties of ceramic materials used in endocrown production [18–20]. With the widespread use of digital workflow, resin matrix ceramic blocks, glass-ceramic blocks developed for subtractive production, and various composite resins produced with the developing additive manufacturing technology are offered to the market. Before clinical application, it is of great importance to comprehensively evaluate the mechanical properties of these newly developed materials through simulation studies that mimic different clinical scenarios [21].

Comprehensive studies comparing the effects of newly introduced materials and manufacturing methods on the fracture resistance of endocrown restorations remain limited. To our knowledge, there is no published study comparing the fracture resistance of the leucid reinforced ceramic (LRC) and PRM used in the current study. To address this gap, the present study aims to compare the fracture resistance of LRC, resin nanoceramic (RNC), lithium disilicate ceramic (LDS) blocks, and printable resin used for endocrowns manufacturing on mandibular molars.

Two hypotheses were tested in this study: The first null hypothesis states that there is no significant difference in the fracture resistance of endocrown restorations fabricated from different materials; the second null hypothesis suggests that the presence of a ferrule does not have a significant effect on the fracture resistance of these restorations.

## Materials and methods

This study was approved by the institutional review board/ethical committee (approval number 2024/24). G*Power v.3.1.9.4 program (Heinrich Heine, Düsseldorf University, Düsseldorf, Germany) was used for statistical analysis in this study. The sample size calculation was based on a previous study [15], which indicated that the sample size for each experimental group should be a minimum of ten (power = 0.90, effect size = 0.5, significance level of α = 0.05). However, to ensure adequate statistical power, the sample size was increased to 12 specimens per group. The materials and their contents used for each group in the study are listed in Table 1.

**Table 1:** The materials and their contents used for each group in the study

PRM		Manufacturer Company	Manufacturing Method	Material	Composition	Lot No
	CROWNTEC	Saremco, Rebstein / Switzerland	Additive Manufacturing	3D-printed ceramic-reinforced composite	Bis-EMA, Dental glass and silica fillers (particle size 0.7 μm, up to 50 wt%), Initiators, Inhibitors and color pigments	E469
LRC	**G CERAM**	ATLAS-ENTA, İzmir/ TÜRKİYE	Subtractive Manufacturing	Leucite-reinforced glass ceramic	65.16% SiO₂, 8.5% Al₂0₃, 3.4% Na₂0, 12.4% K₂O, 0.06% Fe_2_O_3_ 0.02% TiO₂, 0.24% CaO, 0.22% MgO	5608 5598 5683
RNC	**CERASMART**	GC Corp., Tokyo/Japan	Subtractive Manufacturing	Resin nanoceramic	71% silica and barium glass nanoparticles, 29% Bis-MEPP, UDMA, and DMA polymers	2,405,011
LDS	**TESSERA**	Sirona Dentsply, Milford/ USA	Subtractive Manufacturing	Lithium disilicate glass ceramic	90% Li_2_O_5_Si_2_, 5% Li_3_PO_4_, 5% LiAlSi_2_O_6_ (Virgilite)	16,017,786

In the study, 108 mandibular molars that were extracted for periodontal reasons, with homogeneous dimensions and free of fractures, cracks, or caries, were used. Root canals were prepared using the ProTaper Next rotary file system. The mesial canals were sequentially enlarged with files X1 and X2, while the distal canals were prepared using files X1, X2, and X3. Root canal was obturated with gutta-percha points and epoxy resin sealer using the lateral compaction technique. Then canal orifices were sealed with flowable composite resin.

A periodontal ligament simulation was performed for all specimens using a polyether impression material with a thickness of 0.2–0.3 mm [22]. To simulate the bone level, the specimens were embedded in standardized polymethyl methacrylate blocks up to 1 mm below the cementoenamel junction (CEJ).

**Fig. 1:**
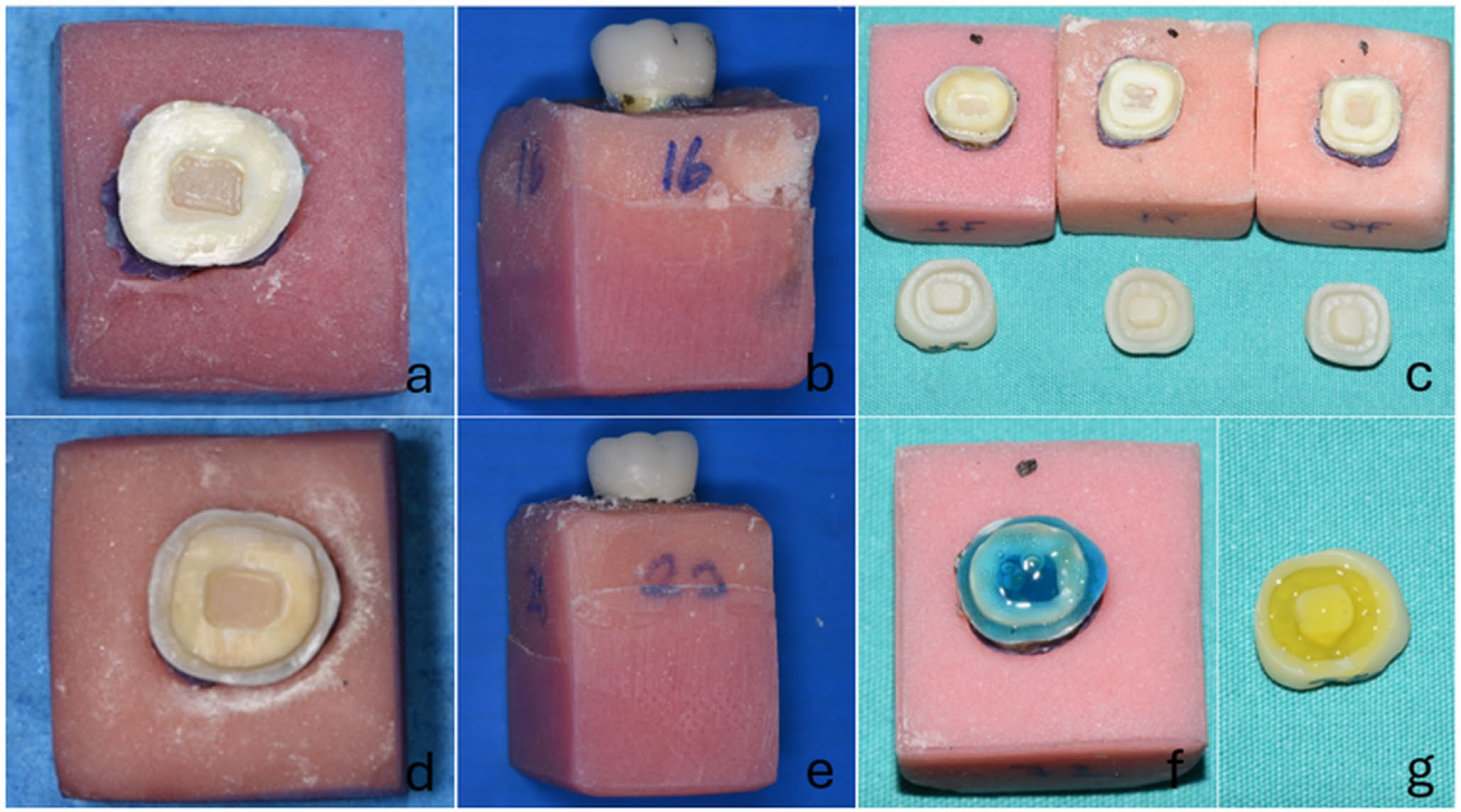
**a**) Preparation of non ferrule specimens, **b**) Cementation of endocrowns in specimens non ferrule, **c**) Ferrule group specimens and endocrowns before cementation, **d**) Preparation of ferrule specimens, **e**) Cementation of endocrowns in specimens ferrule, **f**) Etching of the specimens with 37% orthophosphoric acid, **g**) Etching of the LDS restoration with 5% hydrofluoric acid

For the preparation of the ferrule (F) group teeth, the crowns were sectioned 1 mm above the cemento-enamel junction under water cooling, based on recommendations from previous studies [23, 24]. Subsequently, adhering to the principles of endocrown preparation, a circumferential shoulder-type finish line with a width of 1 mm and a pulp chamber with a depth of 2 mm were prepared (Fig. 1). In the ferrule group, the remaining coronal wall thickness was standardized between 1 and 1.5 mm to ensure uniformity and adequate structural support. For the preparation of the non-ferrule (NF) group teeth, the crowns were removed at the cemento-enamel junction under water cooling, and a pulp chamber with a depth of 2 mm was prepared. All preparations were performed by a single researcher to ensure standardization (Fig. 1).

The digital impressions of the prepared specimens were obtained using an intraoral scanner (Cerec Primescan, Sirona, Bensheim, Germany). The digital data were then exported into the CAD software (inLab CAD SW 22.4, Sirona, Bensheim, Germany). The occlusal form and anatomy of the restorations were determined based on the right mandibular first molar model available in the software database. To ensure standardization of the occlusal surface form, the software-predicted morphology was adapted using the positioning tools, without any distortion of the predicted form. All restorations were designed such that the distance between the cusp tip and the restoration margin ranged from 5.5 to 6 mm, and the distance between the central fossa and the pulpal floor ranged from 3.5 to 4 mm.

**Fig. 2:**
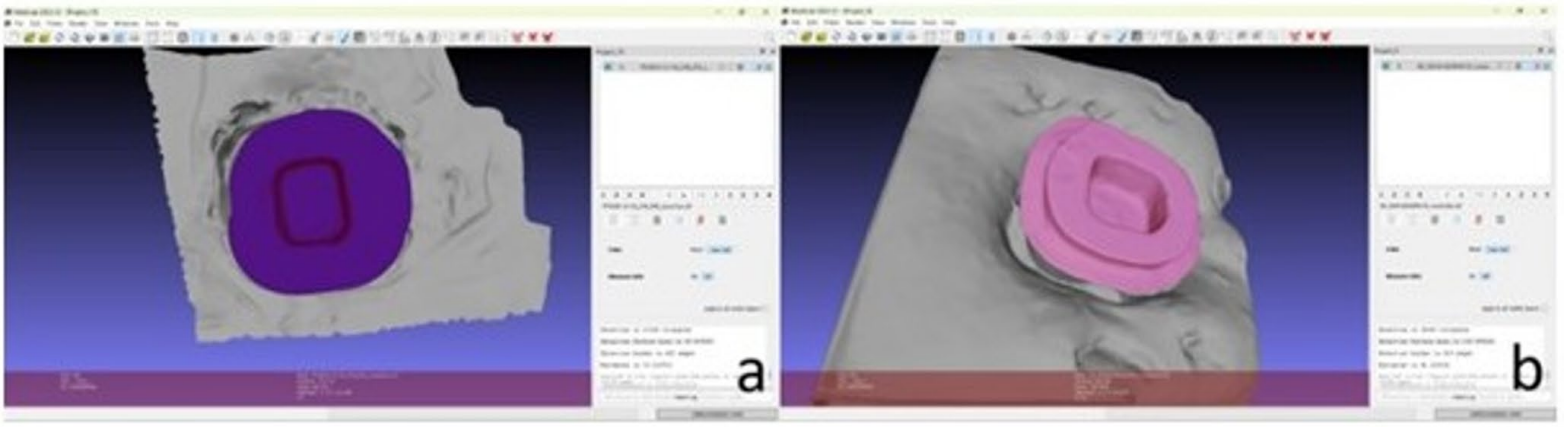
Measurement of the cementation surface area using the MeshLab software. Representative 3D visualizations of the prepared tooth models are shown for (**a**) non-ferrule and (**b**) ferrule specimens

After all these procedures, cementation surface of preparation measurements were performed using the measuring tool of the open-source 3D mesh-processing software MeshLab (MeshLab), as previously applied in similar study [25]. The measurement process and representative visualization are shown in Fig. 2. Subsequently, to achieve an equal distribution of teeth among the groups, specimens with ferrules and non-ferrules were separately randomized, and 108 teeth were divided into nine groups, with the average surface areas of each group as close as possible.

Specimens with 1-mm shoulder-type ferrules were divided into four groups according to the restorative material used for endocrown fabrication;

- **Group LRC -F**: Endocrowns manufactured from LRC blocks (G Ceram)
- **Group RNC-F**: Endocrowns manufactured from RNC blocks (Cerasmart)
- **Group LDS-F**: Endocrowns manufactured from LDS blocks (Tessera)
- **Group PRM-F**: Endocrowns were manufactured using a 3D printer with PRM (Crowntec)

Specimens with non-ferrule were divided into four groups according to the restorative material used for endocrown fabrication;

- **Group LRC-NF**: Endocrowns manufactured from LRC blocks (G Ceram)
- **Group RNC-NF**: Endocrowns manufactured from RNC blocks (Cerasmart)
- **Group LDS-NF**: Endocrowns manufactured from LDS blocks (Tessera)
- **Group PRM-NF**: Endocrowns were manufactured using a 3D printer with PRM (Crowntec)
- **Group Control**: Intact teeth

Restorations were fabricated using a CEREC MCX milling unit (Dentsply Sirona, York, PA, USA) for the LRC, LDS, and RNC blocks. After milling, LDC and LRC restorations were glazed using a calibrated furnace (Speedfire, Dentsply Sirona, York, USA). 5% hydrofluoric acid (Ultradent Porcelain Etch, Ultradent Product Inc., Cologne, Germany) was applied to the bonding surfaces of LDS and LRC restorations for 60 s to achieve surface conditioning according to the manufacturer’s instructions. Following the etching procedure, a silane agent (Ultradent Product Inc., Cologne, Germany) was applied to the treated surfaces and allowed to dwell for 60 s (Fig. 1).

After milling, RNC restorations were glazed with a resin composite glaze (Optiglaze, GC, Tokyo, Japan). After the glazing process, the cementation surface of the RNC restorations was roughened with 30 μm silica-modified aluminum oxide particles (Rocatec Soft, 3 M Oral Care; St Paul, MN, USA) perpendicular to the surface from a distance 10 mm during 10 s with 30 psi pressure according to the manufacturer’s instructions.

PRM restoration was fabricated using an Asiga Max 3D printer (Asiga, Sydney, Australia). The fabrication parameters were set as follows: 50 μm layer thickness, 0.017 s minimum exposure time, 0.01/10.66 mW/cm² minimum/maximum light intensity. After completion of the additive manufacturing process, residual resin on the Crowntec restorations was cleaned using an applicator with alcohol, and post-curing procedures were performed according to the manufacturer’s instructions and previous study. The cleaning procedures were performed according to the manufacturer’s instructions and in line with on previously reported studies [26]. After the post-cure process, the cementation surface of the PRM restorations was roughened by using 110 μm aluminum oxide particles (Korox, Bego, Bremen, Germany) at 1.5 bar perpendicular to the surface from a distance 10 mm during 10 s, and then silane (Ultradent Product Inc., Cologne, Germany) was applied.

During the cementation protocol, the enamel and dentin surfaces of each specimen were conditioned with 37% phosphoric acid (Select HV Etch, Bisco, Schaumburg, IL, USA) for 30 s. The surfaces were then rinsed with an air-water spray for 20 s and dried. A universal adhesive resin (All-Bond Universal, Bisco, Schaumburg, IL, USA) was applied for 20 s and gently air-thinned for 5 s. The manufactured restorations were cemented using a dual-cure resin cement (Duo-Link Universal, Bisco, Schaumburg, USA) following the manufacturer’s instructions. The preparation of the specimens and the restorations after cementation are presented in Fig. 1.

### Fracture load test

**Fig. 3:**
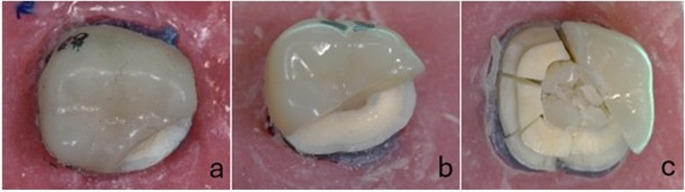
Photographs of the three types of failure modes: **a**. Type I (Restoration fracture), **b**. Type II (Repairable fracture tooth and restoration), **c**. Type III (Non-restorable fracture)

After cementation, fracture resistance testing was performed on the specimens using a universal testing machine (Instron; Instron Corp., MA, USA). Each specimen was positioned on the machine base, and consistent with previous studies [4, 27] an axial load was applied at the center of the occlusal surface using a 5-mm-diameter round stainless steel head, directed perpendicularly to the occlusal plane. The load was applied at a crosshead speed of 1 mm/min. The maximum load at fracture was recorded in Newtons (N). The failure mode of the specimens was classified as follows: Type I, restoration fracture; Type II, restorable fracture involving both the tooth and the restoration; or Type III, non-restorable fracture of the remaining tooth structure [28]. The different types of failures are shown in Fig. 3.

### Statistical analysis

Data were analyzed using the IBM SPSS V23 program (SPSS Inc., Chicago, IL, USA). Data distribution was tested for normality using the Shapiro–Wilk test. For comparison of two groups with normally distributed data, the Independent Samples t-test was used. In addition, independent-samples t-tests were performed to compare the ferrule and non-ferrule subgroups within each restorative material, as the study design aimed to evaluate pairwise differences between material-specific subgroups rather than interaction effects. For three or more groups, one-way ANOVA was applied for normally distributed variables (with post hoc Tamhane’s test), and the Kruskal-Wallis H test was used for non-normally distributed variables (with post hoc Dunn’s test). Relationships between categorical variables were examined with Fisher’s exact test with Monte Carlo correction and the Pearson chi-square test. Descriptive statistics for quantitative variables were presented as mean ± standard deviation, and categorical data were expressed as frequency and percentage. The significance level was set at *p* < .05.

## Results

Table 2 presents the statistical results regarding the surface areas of the specimens randomly assigned to the groups. The difference between the ferrule and non-ferrule groups in terms of the surface areas of the allocated teeth was statistically significant (*P* < .05), whereas the differences within the ferrule groups themselves and the non-ferrule groups were not statistically significant.

**Table 2:** Comparison of surface area values according to groups and ferrule status

LRC	Ferrule	Non Ferrule		*p* ^x^	η^2^
	126,58 ± 8,85^A^	102,19 ± 6,79^B^	Group	0,467	0,028
RNC	126,2 ± 14,55^A^	98,68 ± 13,95^B^	Ferrule status	**< 0**,**001**	0,608
LDS	125,61 ± 13,72^A^	104,95 ± 13,67^B^	Group x Ferrule status	**0**,**016**	0,11
PRM	138,94 ± 12,94^A^	96,88 ± 9,14^B^			
Total	129,33 ± 13,52	100,67 ± 11,38			

^x^Two Way ANOVA; Mean+ Std. Deviation, A−B: Same superscript letters identify statistically similar groups

η^2^: Partial eta squared, LRC: Leucite Reinforced Ceramic, RNC: Resin Nanoceramic, LDS: Lithium Disilicate Ceramic, PRM: Printable Resin Material

Table 3 summarizes the descriptive statistics, including the mean and standard deviation, of the fracture resistance values for all groups according to the presence or absence of a ferrule. A statistically significant difference was observed in the mean fracture resistance values among the materials in the groups (*P* < .05). Independent of the presence of a ferrule, the Control group exhibited the highest fracture resistance, whereas the LRC group demonstrated the lowest. In the comparison of the ferrule groups, a statistically significant difference was observed (*P* < .05). The differences between the Control, PRM-F, RNC-F, and LDS-F groups and the LRC-F group were found to be statistically significant (*P* < .05), whereas no significant difference was detected among the Control, PRM-F, RNC-F, and LDS-F groups. Nonetheless, the fracture resistance values of all endocrown groups were determined to exceed the maximum masticatory forces reported in the literature [29].

**Table 3:** Intragroup comparison of fracture resistance of Ferrule, Non-Ferrule groups according to material type

LRC	Ferrule (F) Mean ± SD (*N*)	Non-Ferrule (NF) Mean ± SD (*N*)
	805,11 ± 351,24 ^B^	970,3 ± 399,72 ^F^
RNC	1957,18 ± 825,17 ^A^	1518,51 ± 447,03^D, E^
LDS	1604,15 ± 428,41 ^A^	1309,25 ± 274,96 ^E, F^
PRM	1757,9 ± 352,11 ^A^	1934,27 ± 315,2 ^D^
Control	2259,33 ± 824,62 ^A^	2259,33 ± 824,62 ^D^
	p^x^ **<0**,**001**	p^x^ **<0**,**001**

^x^One Way ANOVA, Mean + Std. Deviation;

*LRC: Leucite Reinforced Ceramic. RNC: Resin Nanoceramic, LDS: Lithium Disilicate Ceramic, PRM: Printable Resin Material, F: Ferrule, NF: Non Ferrule, N: Newton

**Different superscript letters indicate statistically significant differences in each column

In the comparison of the non-ferrule groups, a statistically significant difference was observed (*P* < .05). The differences between the Control, PRM-NF, and RNC-NF groups and the LRC-NF group, as well as between the Control and PRM-NF groups and the LDS-NF group, were found to be statistically significant. Among the ferrule groups, statistically significant differences were also observed between the Control, PRM-F, RNC-F, and LDS-F groups and the LRC-F group.

Table 4 summarizes the comparison of fracture resistance values between the ferrule and non-ferrule groups. Comparisons of ferrule and non-ferrule groups fabricated from the same material revealed no statistically significant differences (*P* > .05 for all paired comparisons).

**Table 4:** Comparison of the fracture resistance values between the ferrule and non-ferrule groups

LRC	Ferrule (F) Mean ± SD (*N*)	Non Ferrule (NF) Mean ± SD (*N*)	*p* ^x^
	805,11 ± 351,24	970,3 ± 399,72	0,294
RNC	1957,18 ± 825,17	1518,51 ± 447,03	0,124
LDS	1604,15 ± 428,41	1309,25 ± 274,96	0,057
PRM	1757,9 ± 352,11	1934,27 ± 315,2	0,209
CONTROL	2259,33 ± 824,62	2259,33 ± 824,62	1,000

^x^ Independent Samples T-Test, Mean + Std deviation;

LRC: Leucite Reinforced Ceramic, RNC: Resin Nanoceramic, LDS: Lithium Disilicate, PRM: Printable Resin Material, F: Ferrule, NF: Non Ferrule, N: Newton

**Fig. 4:**
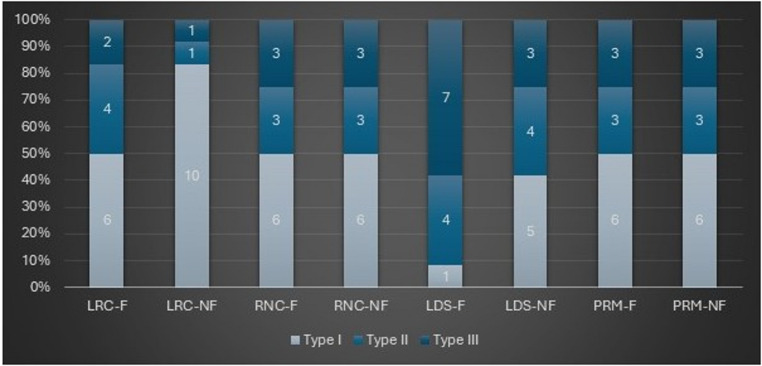
The distributions of failure types in both non-ferrule and ferrule groups according to material groups are illustrated

Moreover, no statistically significant difference was detected in the distribution of fracture types among either the non-ferrule groups (*P* > .05) or the ferrule groups (*P* > .05). The distributions of fracture types in both ferrule and non-ferrule groups, according to material groups, are compared and presented in Fig. 4. Although no statistically significant differences were identified among the materials concerning fracture types, the results indicated that LRC restorations, which exhibited the lowest fracture resistance, were associated with the highest incidence of type I fractures. Restorations fabricated from RNC and PRM materials predominantly demonstrated restorable fractures, namely type I and type II. By contrast, the LDS group exhibited the highest incidence of catastrophic fractures.

## Discussion

Molar teeth are the first permanent teeth to erupt in the oral cavity, and due to their anatomical features, such as the presence of fissures that are difficult to clean, and their posterior location, they are challenging to maintain in terms of oral hygiene [30]. These factors contribute to a higher incidence of caries in molars and a greater need for endodontic treatment compared to other teeth. In the present study, endocrown restorations fabricated from different materials were evaluated in terms of fracture resistance and fracture patterns following preparations of molar teeth with and without a ferrule.

Increasing ferrule height has been shown to increase the fracture resistance of endodontically treated teeth [23, 31]. However, achieving this height under clinical conditions is often not feasible. Therefore, the present study aimed to compare situations with and without a ferrule. Furthermore, the findings of this study are expected to guide clinicians in the selection of appropriate restorative materials and preparation designs, thereby contributing to the long-term success and survival of endodontically treated teeth.

There are contradictions among studies evaluating the effect of periodontal ligament simulation on fracture resistance [32–34]. Some studies have reported that the presence of an artificial PDL may influence fracture modes and increase resistance to fracture [33, 34], whereas others have found no significant effect of PDL simulation on fracture resistance [32]. In the current study, periodontal ligament simulation was preferred to better simulate the real tooth behavior against masticatory forces.

There are studies in the literature that separately compare the mechanical properties of restorations fabricated from different LDC, RNC, and LRC materials [1, 19, 20, 35–38]. Endocrown restorations fabricated from RNC materials have been shown to outperform LDS-based restorations in terms of fracture resistance in one study [39]. This was attributed to the elastic modulus of RNC being closer to that of dentin. However, in the current study, no significant difference was found between the RNC and LDS groups, and these findings are consistent with those reported by Acar et al. [19]. The discrepancy between the results is thought to stem from methodological differences such as the direction of force application and the design of tooth preparation.

LRC showed lower fracture resistance than LDS in a study evaluating their physico-mechanical characteristics [37]. This has been attributed to the nature of the reinforcing agents within the material and its modulus of elasticity, which is significantly higher than that of dentin [35, 36, 38]. The results of the current study are consistent with these findings in the literature. The first null hypothesis, which stated that no difference would be observed in the fracture resistance of endocrowns fabricated from different materials, was rejected.

Additive manufacturing technologies have recently emerged as viable alternatives to subtractive CAD-CAM methods for producing definitive restorations. Compared with milling, 3D printing enables a more material-efficient workflow with reduced initial equipment and consumable costs, while also allowing the simultaneous fabrication of multiple restorations in a single printing cycle. However, it requires additional post-processing steps, such as cleaning and light polymerization, and handling of unpolymerized resin, which can be less convenient in clinical settings [21].

PRM, which are introduced to the market as alternatives to CAD-CAM blocks, are also evaluated in the current study, and PRM demonstrated higher fracture resistance than LRC. This difference can be attributed to both the variations in manufacturing techniques and the mechanical properties of the materials. During milling, microcracks may develop within the glass matrix of LRC materials, whereas such defects do not occur in additive manufacturing. In substractive manufacturing processes, circular burs interact with the material surface at precisely calculated planes and contact points. The advancement of these burs generates characteristic tool marks as a result of the scraping action. Although such macroscopic surface irregularities do not necessarily lead to stress concentration zones, microscopically observed sharp cracks and scratches can act as critical sites for stress accumulation and contribute significantly to material failure [40]. The effect of production method on outcomes was also evident in the LDC group. Additionally, PRM (4 GPa) has a modulus of elasticity much closer to that of dentin compared to LRC (58 GPa) and LDS (85.77 GPa) materials, which may explain the superior fracture resistance observed in the group PRM [9, 41, 42]. Furthermore, considering fracture toughness as a key mechanical parameter, leucite-reinforced ceramics (LRC) are inherently more brittle and exhibit lower resistance to crack initiation and propagation compared with resin nanoceramics [43]. This lower toughness, combined with the brittle glassy microstructure of LRC, facilitates the formation of microcracks both during the milling process and under subsequent mechanical loading, supporting the higher crack density observed in these materials.

There is only a limited number of PRMs available for the fabrication of definitive restorations. Mechanical properties of PRMs have been compared with those of various materials such as RNC, LDC, and PMMA. Some studies reported no significant difference in fracture resistance between PRM and RNC crowns [15, 21, 26], while others found higher fracture resistance for PRM [16, 44], and some for RNC blocks [13, 17]. These conflicting findings have been attributed to various factors, including differences in filler content, production method, monomer structure, sample type, aging protocols, the use of natural teeth or analogues, and variations in production parameters [13, 16, 17, 45, 46].

In the current study, no significant difference was found between the RNC, PRM, and Control groups. This result is consistent with some previous studies on PRM crowns [15, 21, 26]. The lack of difference between RNC and PRM restorations may be due to both materials having a resin-based matrix, despite differences in their filler composition [9, 47].

Skupien et al. [48] reported that the presence of a ferrule improves the prognosis of teeth. Therefore, in cases where a ferrule is not present, the compatibility of the restorative material’s modulus of elasticity with that of dentin may be a critical factor for success. Additionally, the application of ferrule preparation increases the cementation surface area. Hayes et al. [49] compared the fracture resistance and failure types of endocrown restorations prepared with varying pulp chamber cavity depths. Their study concluded that the group with the greatest cavity depth, and consequently the largest cementation surface area, exhibited higher fracture resistance [49]. Similarly, Einhorn et al. reported that specimens prepared with a 1 mm ferrule exhibited a 37% increase in cementation surface area compared to the non-ferrule group [23]. Another notable aspect of the current study is the impact of ferrule presence on the performance of LDS endocrowns. While LDS restorations with a ferrule exhibited fracture resistance similar to the PRM and Control groups, a statistically significant difference was observed between the non-ferrule groups. In the current study, the lower fracture resistance observed in the non-ferrule LDS restorations may be attributed to the reduced cementation surface area and the consequent diminished force distribution.

Nevertheless, in the current study, no statistically significant difference was observed between the ferrule and non-ferrule specimens within the restorative material groups, and thus, the second null hypothesis was accepted. This outcome aligns with the findings of Rocca et al. [4], which may be attributed to the use of similar tooth preparation designs in both studies. This finding may be related to the fact that the thickness and overall strength of the restorative materials could have compensated for the differences in cavity design between the ferrule and non-ferrule groups.

Although the ideal ferrule height has been widely reported to be 1.5–2.0 mm [48, 50], recent experimental evidence suggests that a minimal ferrule height of 1.0 mm may still provide a biomechanically effective reinforcement, particularly in severely compromised teeth [50]. Meng et al. reported that the ideal ferrule height for restorations of endodontically treated teeth was 2.0 mm; however, no statistically significant difference was observed between the 1.0 mm and 2.0 mm ferrule groups [50]. Based on these findings, the present study intentionally adopted a 1 mm ferrule height to simulate clinically realistic conditions in teeth with extensive structural loss, where achieving a 2 mm ferrule may not always be feasible. This minimal ferrule height was deliberately selected based on previous evidence indicating that comparable fracture resistance can be achieved under such conditions [23, 24]. Further in vitro studies with larger sample sizes and higher ferrule heights are recommended to clarify the influence of ferrule height on fracture resistance.

In clinical practice, it is important to determine whether the remaining tooth structure is restorable after a failure occurs [28]. In the current study, no statistically significant difference was observed in the failure distributions; however, this may be attributable to the small sample size (*n* = 12). Moreover, the highest rate of catastrophic fractures was observed in the LDS group. This may be attributed to the fact that LDS had the highest modulus of elasticity among the materials tested in this study. These results were in agreement with other studies, which were related to the difference in modulus of elasticity between the materials [1, 20]. Lithium disilicate has a high elastic modulus compared to natural dentin and is structurally quite rigid [1, 18, 51]. As a result, the restoration-tooth complex becomes more susceptible to catastrophic failure [1, 51].

Intrinsically, the limitations of this study are that only the fracture strength and failure type were considered, the number of specimens per group (*n* = 12), application of force only in the axial direction, and the absence of thermomechanical aging. Further studies should include in vivo studies with a large group of specimens, testing the competence of these restorations in the oral cavity.

## Conclusions

Within the limitations of this in-vitro study, it can be inferred that the materials evaluated demonstrated different fracture resistance behaviors depending on their microstructural characteristics and manufacturing techniques. The resin matrix-based RNC and PRM groups, as well as the LDS group, exhibited higher fracture resistance compared to the LRC group. Furthermore, while LRC, which showed lower fracture resistance, generally resulted in restorable fractures, LDS restorations tended to show catastrophic failures. On the other hand, PRM and RNC groups exhibited higher fracture strength and predominantly restorable failure modes. However, since no artificial aging or dynamic loading was applied and only axial forces were tested, these findings should be interpreted with caution, as in-vitro conditions cannot fully replicate the complex mechanical and environmental factors present in the oral cavity.

## Data Availability

No datasets were generated or analysed during the current study.
